# Dietary behaviors, nutrition-monitoring technology use, and their socio-demographic determinants among Moroccan adults: a cross-sectional study

**DOI:** 10.3389/fnut.2026.1912937

**Published:** 2026-09-10

**Authors:** Morad Kaddouri, Salah Chaji, Omar Ait El Alia, Zakaria Asbai, Abdelaziz Ait Melloul, Morad Oubouali, Nouhaila Ajbli, Sana El Fadeli, Abdelaaty A. Shahat, Rashed N. Herqash, Joe Miantezila Basilua, Khalid Boutoial

**Affiliations:** 1 Laboratory of the Engineering and Applied Technologies, Higher School of Technology, Sultan Moulay Slimane University, Beni Mellal, Morocco; 2 Department of Drug Science and Technology, University of Turin, Turin, Italy; 3 Laboratory of Biochemistry, Agri-Food Materials and Environment, Hassan 2 University of Casablanca, Mohammedia, Morocco; 4 Laboratory of Water Sciences, Microbial Biotechnologies and Natural Resources Sustainability (AQUABIOTECH), Unit of Microbial Biotechnology, AgroSciences and Environment (BIOMAGE)-CNRST Labeled Research Unit N° 4, Faculty of Sciences Semlalia, Cadi Ayyad University (UCA), Marrakech, Morocco; 5 Laboratory of Computer Mathematics and Modeling of Complex Systems, Higher School of Technology of Essaouira, Cadi Ayyad University, Essaouira, Morocco; 6 Department of Pharmacognosy, College of Pharmacy, King Saud University, Riyadh, Saudi Arabia; 7 Center for Research on Medicinal, Aromatic, and Poisonous Plants, DSR, King Saud University, Riyadh, Saudi Arabia; 8 Department of Biostatistics and Mathematics, Faculty of Pharmacy, Paris Cité University, Paris, France

**Keywords:** cross-sectional study, dietary behaviors, digital health, Mediterranean diet, Morocco, nutrition monitoring, nutrition transition, obesity

## Abstract

**Problem considered:**

In our sample of urban, educated Moroccan adults, we observed dietary patterns suggestive of a nutrition transition, characterized by high consumption of both traditional healthy foods and processed, energy-dense items. This study assessed dietary practices, dieting habits, and use of nutrition-monitoring technologies among Moroccan adults, and examined socio-demographic associated factors.

**Methods:**

A cross-sectional online survey was conducted from October 2024 to January 2025 among 1,035 Moroccan adults. A structured questionnaire collected data on socio-demographics, dietary behaviors, dieting history, and use of nutrition monitoring technologies. BMI was calculated from self-reported weight and height. Associations were tested using chi-square analysis with Bonferroni correction for multiple comparisons; multivariable multinomial logistic regression was also performed to assess independent associations.

**Results:**

Participants were predominantly urban (82.9%) and university-educated (79.8%). Overweight/obesity prevalence was 60.9%. Fresh fruit (89.7%) and vegetable (89.0%) consumption was high, but high-fat foods (76.6%) and sweetened beverages (49.1%) were also widely consumed. Half (49.6%) had previously dieted, mainly sugar-free or low-calorie, yet only 9.5% used digital tools for dietary monitoring. After Bonferroni correction, significant associations remained for adding salt after cooking with education (*p* < 0.001) and consuming fried foods with education (*p* < 0.001), and trying to reduce sugar intake with age (*p* < 0.001). In multivariable multinomial regression, gender was the most consistent independent predictor of dietary behaviors; age, education, and income showed select independent associations. No association was found between BMI and dietary habits.

**Conclusion:**

In our sample of urban, educated Moroccan adults, we observed dietary patterns that suggest a co-existence of traditional healthy foods with unhealthy items. Overweight/obesity is highly prevalent in this population subgroup. Despite interest in dieting, use of nutrition-monitoring technology remains very low in our sample. Hypothesis-generating suggestions include culturally adapted digital tools and targeted interventions, but confirmatory studies using probability sampling are needed.

## 1 Introduction

The traditional Moroccan diet is characterized by a rich diversity of plant-based foods, including fresh fruits and vegetables, legumes, whole grains, and olive oil, complemented by moderate consumption of fish, poultry, and dairy products, with limited red meat intake (1, 2). This dietary pattern shares many features with the Mediterranean diet (MeDi), which is widely recognized as a healthy eating pattern linked to lower risk of chronic diseases and better health outcomes (3, 4). The MeDi is defined by high consumption of plant-based foods, olive oil as the primary fat source, moderate fish and poultry consumption, and low intake of red meat and processed foods (5). In Morocco, the traditional diet has historically aligned with these Mediterranean principles, with regional variations incorporating local culinary traditions and agricultural products (6, 7).

However, Morocco has experienced changes in dietary patterns in recent decades, consistent with global nutrition transition patterns, driven by rapid urbanization, economic development, and shifts in lifestyle and dietary patterns (8, 9). These changes have been characterized by increased consumption of processed and ultra-processed foods, sweetened beverages, and high-fat products, alongside decreased intake of traditional staples such as legumes, whole grains, and fresh produce (9, 10). A previous study by El Rhazi et al. (11) showed that adherence to the traditional MeDi was already low among Moroccan adults a decade ago. Lower adherence was linked to living in rural areas, being single, divorced, or widowed, and having obesity. No significant links were found with age, gender, or education (11).

Since then, factors such as globalization, digitalization, and socio-economic changes have likely accelerated these dietary transitions. The growing availability of processed foods, new dietary trends (e.g., gluten-free, low-carb), and digital health tools are now shaping food choices (9). At the same time, rising rates of obesity and non-communicable diseases (NCDs) in Morocco highlight the urgent need to better understand current eating habits and their determinants (12).

However, previous Moroccan studies (1, 9, 11, 12), have several limitations: (1) they were conducted before the widespread adoption of digital technologies and social media in Morocco; (2) they focused primarily on adherence to the traditional Mediterranean diet rather than examining contemporary dietary practices influenced by globalization; (3) they did not assess the use of nutrition-monitoring technologies, which are increasingly available globally but whose adoption in Morocco remains unexplored; and (4) they did not specifically examine the interplay between dietary behaviors and socio-demographic factors in a predominantly urban, educated population a demographic segment particularly exposed to dietary transitions and potentially receptive to digital health interventions. To our knowledge, no study to date has simultaneously examined dietary behaviors, dieting practices, and technology use for nutrition monitoring in the Moroccan context.

This study specifically focuses on young, urban, and highly educated Moroccan adults for several compelling reasons. First, urban populations are at the forefront of dietary transitions, as they have greater exposure to globalized food systems, processed foods, and fast-food culture, while simultaneously experiencing lifestyle changes (sedentary work, reduced physical activity) that promote obesity (13). Second, younger adults (18–44 years) represent the demographic segment most affected by the nutrition transition, as they are the primary adopters of new dietary trends and are heavily influenced by social media marketing of processed foods (14). Third, educated individuals often serve as early adopters of health technologies and are more likely to engage with digital health tools, making them a key target population for technology-based interventions (15, 16). Fourth, this population subgroup is underrepresented in nutritional research in Morocco, with most previous studies focusing on either general populations or specific clinical groups, leaving a gap in understanding the dietary behaviors of this emerging demographic segment (1, 9, 11, 12). Fifth, understanding the dietary patterns and technology adoption behaviors of this group is essential for designing targeted public health interventions that leverage digital platforms to promote healthy eating, as this population demonstrates both the highest potential for behavior change and the greatest access to digital resources (17). By examining this specific subgroup, we aim to generate evidence that can inform the development of culturally adapted digital health interventions for populations at the forefront of dietary change.

This study aims to address these identified gaps by: (1) describing current dietary patterns in a contemporary Moroccan sample; (2) measuring the frequency of specific food consumption and adherence to popular modern diets; (3) evaluating the use of technology for health monitoring; and (4) explore associations between dietary behaviors and socio-demographic factors using both bivariate and multivariable analytical approaches. Given the descriptive exploratory nature of this study, we present both unadjusted (chi-square) and adjusted (multivariable multinomial logistic regression) analyses to provide a comprehensive examination of potential associations while acknowledging the exploratory nature of the findings.

Based on the literature and the Moroccan context, we formulated the following hypotheses:

*H1*: Despite high education levels, the prevalence of overweight/obesity will remain elevated in this urban population, reflecting the impact of obesogenic environments;

*H2*: A ‘dual burden’ dietary pattern will coexist, with traditional healthy foods (fruits, vegetables) consumed alongside processed, energy dense items (high-fat foods, sweetened beverages);

*H3*: Gender, age, and education will be the most significant socio-demographic predictors of dietary behaviors, consistent with findings from other transitioning economies;

*H4*: The use of nutrition monitoring technology will be low despite a stated interest in dietary modification, indicating a potential gap in digital health adoption.

By comparing our results with earlier data, this study will help track changes in Moroccan dietary habits over the past decade and support the development of targeted public health interventions in the 21st century.

## 2 Materials and methods

### 2.1 Ethical statement

All procedures involving human beings have been reviewed and approved by the Research Ethics Committee of Sultan Moulay Slimane University. The information was obtained with the voluntary and informed consent of the participants. Additionally, the data collection tool used (questionnaire) has the advantage of being impersonal since the information obtained cannot be linked to the participants and the subjects. Therefore, this approach is safer in terms of anonymity, confidentiality of information and respect for the privacy of participants.

### 2.2 Study population, sample size, and data collection

The targeted population for the study consisted of Moroccan individuals. Eligibility for participation was based on the following criteria:

- Inclusion criteria: (1) Adults aged 18 years or older; (2) Moroccan nationality (or long-term resident, defined as >12 months); (3) Able to read and understand Arabic or French (the languages in which the questionnaire was administered); (4) Access to internet and a smartphone, tablet, or computer to complete the online survey; (5) Voluntary consent to participate in the study.
- Exclusion criteria: (1) Individuals under 18 years of age; (2) Individuals not residing in Morocco at the time of survey completion; (3) Individuals currently hospitalized or institutionalized; (4) Individuals with diagnosed eating disorders (self-reported), as their dietary behaviors may differ significantly from the general population; (5) Pregnant or lactating women, as their nutritional needs and dietary behaviors may differ from the general population; (6) Individuals who were unable to complete the questionnaire due to literacy barriers or visual impairment; (7) Non-residents or tourists visiting Morocco.

These criteria were applied at the beginning of the online questionnaire, where potential participants were presented with the study information and asked to confirm their eligibility before proceeding. Ineligible individuals were directed to a thank-you page and did not have access to the full questionnaire.

A convenience sampling approach was employed, which was deemed appropriate for this exploratory study given the following considerations: (1) the lack of a comprehensive sampling frame for the Moroccan adult population with accessible contact information, as no national database of email addresses or phone numbers is publicly available; (2) the study’s focus on digitally literate individuals who would be potential users of nutrition monitoring technologies this represents the primary target population for future digital health interventions; (3) the practical constraints of conducting a large scale survey within a limited timeframe (4 months) and without external funding for fieldwork, which precluded probability sampling methods requiring extensive resources; and (4) the desire to reach a geographically dispersed sample across Morocco, which is facilitated by digital recruitment platforms.

Recruitment was conducted through a multi-channel approach to maximize reach. The survey link was distributed through the following channels:

- *Institutional networks*: the research team disseminated the survey through official university email lists at Sultan Moulay Slimane University, Hassan II University of Casablanca, and Cadi Ayyad University, reaching faculty members, staff, and students. Additionally, the survey was shared through professional networks of the research team members, including public health associations and nutrition professional groups.
- *Social media platforms*: the survey link was posted on public Facebook groups focused on health, nutrition, and lifestyle topics; shared via Instagram stories and posts through the researchers’ institutional and personal accounts; and distributed through WhatsApp groups of academic cohorts, professional networks, and community groups. The researchers also collaborated with social media influencers in the health and wellness space to share the survey with their followers.
- *Snowball sampling*: participants were encouraged to share the survey link with their contacts (e.g., family, friends, colleagues) through forwarding the WhatsApp message or reposting on their social media accounts.
- *Targeted outreach*: the research team directly contacted specific organizations, including community centers, health clinics, and professional associations, requesting that they forward the survey link to their members.

The recruitment messages provided detailed information about the study objectives, the voluntary nature of participation, the estimated completion time (approximately 10–12 min), and included a direct link to the secure online questionnaire hosted on Google Forms. No monetary or material incentives were offered for participation, and participants were informed that their responses would be used exclusively for academic research purposes. To avoid duplicate responses, participants were required to complete the survey only once, and the Google Forms platform was configured to prevent multiple submissions from the same IP address. Of the 1,245 individuals who accessed the questionnaire, 1,035 completed all sections, yielding a completion rate of 83.1%.

Nevertheless, we acknowledge the inherent limitations of this sampling strategy. The sample over-represents urban residents (82.9%), university-educated individuals (79.8%), and younger adults (32.3% aged 18–24, 67.1% aged 25–64), while under-representing rural populations, those with lower educational attainment, and older adults (only 0.6% aged ≥65 years). This composition reflects both the recruitment channels used and the digital divide that persists in Morocco. Consequently, findings should be interpreted as applying primarily to urban, educated, digitally connected Moroccan adults and cannot be generalized to the entire Moroccan adult population without further validation studies using probability sampling methods.

Anthropometric data (weight and height) were self-reported through categorical responses in the online questionnaire by selecting the corresponding pre-defined groups. Body Mass Index (BMI) was subsequently calculated for analytical purposes by taking the midpoint of each weight and height category (e.g., 65.5 kg for the 61–70 kg group, 175.5 cm for the 171–180 cm group) and using the standard formula (weight (kg)/height (m^2^)). BMI was classified according to the World Health Organization (WHO) guidelines: underweight (<18.5 kg/m^2^), normal weight (18.5–24.9 kg/m^2^), overweight (25–29.9 kg/m^2^), and obese (≥30 kg/m^2^). A total of 1,035 responses were collected, which was considered an adequate sample size for the scope of the study.

### 2.3 Sample size calculation

The required sample size was calculated using Cochran’s formula; n_0_ = *Z^2^pq*/e^2^, where n_0_ = initial sample size estimate, *Z* = 1.96 at a 95% confidence level, e = margin of error (5% or 0.05), and q = 1 − p. with *p* = 50% was used. Subsequently, the following modified Cochran’s formula was used for calculating the adjusted sample size in a small population: n = *n_0_N*/[*N* + (*n_0_*–1)]; here, n = adjusted sample size, *n_0_* = 385 (from initial calculation), and N represented the total population size, which was 36,828,330 inhabitants in 2024, According to the 2024 Population and Housing Census in Morocco (18). The minimum sample size for this calculation was 385. Although the study used a non-probability convenience sampling method (which does not strictly require a sample size calculation), this calculation was performed for descriptive purposes to provide a reference. The obtained sample (*n* = 1,035) exceeds the calculated minimum, which supports the descriptive power of the study while acknowledging that generalizability is limited by the sampling strategy.

### 2.4 Questionnaire design and outcome measures

The questionnaire was developed by adapting items from three validated instruments: the NHANES Dietary Behavior Questionnaire, the Mediterranean Diet Adherence Screener (MEDAS), and the WHO STEPS Instrument (19–21). A forward-backward translation process was conducted from English into Arabic and French. Content validity was assessed by a panel of five Moroccan experts (nutritionists, epidemiologists, public health specialists), yielding a Scale-Level Content Validity Index (S-CVI) of 0.89. The questionnaire was pilot-tested on 50 Moroccan adults to assess comprehension and clarity, resulting in minor wording modifications. Internal consistency for the dietary practices section (8 items) was acceptable (Cronbach’s *α* = 0.78). The final questionnaire included 19 items grouped into three sections: (a) sociodemographic characteristics (8 items), (b) dietary practices (8 items with 5-point Likert scale, Never, Rarely, Sometimes, Often, Always), and (c) dieting history and technology use (3 items).

The questionnaire was developed by adapting items from three validated instruments: the NHANES Dietary Behavior Questionnaire (19), the Mediterranean Diet Adherence Screener (MEDAS) (20), and the WHO STEPS Instrument (20, 21). The rationale for item selection was as follows:

- *From NHANES*: we selected items assessing the frequency of consumption of key food groups (fruits, vegetables, sweetened beverages, fried foods, high-fat foods, fiber-rich foods) because these items capture core dietary behaviors relevant to the nutrition transition and have established psychometric properties. Items related to specific nutrient intake (e.g., vitamin D, calcium) were excluded as they were beyond the scope of this study.
- *From MEDAS*: we retained items related to healthy eating practices (fruit and vegetable consumption, fiber intake) while excluding more specific Mediterranean diet components (e.g., olive oil consumption, nut intake) to focus on general dietary behaviors.
- *From WHO STEPS*: we adapted items on salt intake and sugar reduction as these behaviors are directly relevant to non-communicable disease prevention in Morocco.

We deliberately shortened the scales (from the original ~40 items to 8 dietary practice items) to reduce respondent burden and improve completion rates, while maintaining content validity. This approach was deemed appropriate for our research objectives, which focused on frequency of consumption rather than detailed dietary assessment.

A forward-backward translation process was conducted from English into Arabic and French by two independent bilingual translators, with discrepancies resolved through consensus. Content validity was assessed by a panel of five Moroccan experts (nutritionists, epidemiologists, public health specialists) who rated the relevance of each item. The Scale-Level Content Validity Index (S-CVI) was 0.89, exceeding the acceptable threshold of 0.80. The questionnaire was pilot-tested on 50 Moroccan adults (25 Arabic-speakers, 25 French-speakers) to assess comprehension, clarity, and face validity, resulting in minor wording modifications. Internal consistency for the dietary practices section (8 items) was acceptable (Cronbach’s *α* = 0.78). The final questionnaire included 19 items grouped into three sections: (a) sociodemographic characteristics (8 items), (b) dietary practices (8 items with 5-point Likert scale: Never, Rarely, Sometimes, Often, Always), and (c) dieting history and technology use (3 items).

### 2.5 Statistical analysis

Data were extracted from Google Forms into Microsoft Excel for cleaning and organization. All statistical analyses were performed using IBM SPSS Statistics version 28.0. A *p*-value of less than 0.05 was considered statistically significant for all tests. Given the multiple chi-square tests performed in this study (8 dietary behaviors × 6 sociodemographic factors = 48 tests), we applied the Bonferroni correction for multiple comparisons to control the family-wise error rate at *α* = 0.05. The adjusted significance level was calculated as α_adj = 0.05/48 = 0.00104. Thus, only associations with *p* < 0.001 were considered statistically significant after correction. Associations with 0.001 ≤ *p* < 0.05 are reported as nominally significant and are interpreted with caution, acknowledging the potential for Type I errors.

For all chi-square analyses, the assumptions of the test were checked prior to interpretation. The minimum expected cell frequency for all analyses was verified to ensure that it exceeded 5, the standard threshold for chi-square validity. In cases where expected frequencies were below 5, categories were combined to meet this assumption. A summary of the minimum expected cell frequencies for each analysis is reported in Supplementary Table S3.

To assess the independent effects of sociodemographic factors on each dietary behavior (ordinal variable with 5 levels), we conducted multinomial logistic regression analyses for each of the eight dietary behaviors. Each model included age, gender, education, residence, and income as independent variables, with ‘Often/Always’ as the reference category. This approach allows for the simultaneous adjustment of multiple confounders and provides a more robust assessment of independent associations compared to bivariate chi-square analyses. Adjusted odds ratios (OR) with 95% confidence intervals (CI) were calculated for each predictor variable in each dietary behavior model. Model fit was assessed using AIC and BIC, and the proportional odds assumption was tested using the Brant test when applicable.

In addition to bivariate chi-square tests, a multivariable binary logistic regression was performed to identify factors associated with overweight/obesity (BMI ≥ 25 vs. < 25). The model included age group, gender, education level, residence, monthly income, and all eight dietary behavior scores (each treated as continuous variables from 1 to 5). Variables were entered into the model using a simultaneous (forced entry) approach based on: (a) theoretical relevance derived from the literature, (b) statistically significant bivariate associations (*p* < 0.10), and (c) clinical importance regardless of statistical significance in bivariate tests. This approach was chosen over stepwise selection to avoid data-driven variable selection and to ensure the inclusion of clinically relevant variables.

Prior to model estimation, multicollinearity among the eight dietary behavior variables (each scored from 1 to 5) was assessed using Variance Inflation Factors (VIF) and tolerance values. The VIF values ranged from 1.21 to 1.86 (mean VIF = 1.45), all well below the conventional threshold of 10 indicating no problematic multicollinearity. Similarly, tolerance values ranged from 0.54 to 0.83, all exceeding the threshold of 0.2. These diagnostics confirm that the dietary behavior variables can be included simultaneously in the regression model without concerns about inflated standard errors or unstable coefficient estimates. Adjusted odds ratios (OR) with 95% confidence intervals (CI) were calculated, and model fit was assessed using AIC, BIC, and McFadden’s pseudo-*R*^2^.

## 3 Results

### 3.1 Socio-demographic characteristics of the study population

A total of 1,035 participants completed the study. The socio-demographic and anthropometric characteristics of the sample are presented in Table 1. The majority of participants were female (52.3%), and the mean age was predominantly within the 25–64 years category (67.1%). The sample was highly urbanized (82.9%) and reported a high level of education, with 79.8% holding a bachelor’s degree or higher. Over half of the participants (53.4%) reported a monthly income of less than 4,000 MAD. Based on self-reported weight and height, the calculated Body Mass Index (BMI) distribution indicated that 60.9% of the participants were overweight or obese (BMI ≥ 25 kg/m^2^).

**Table 1:** Socio-demographic characteristics of the study participants, overall and by gender (*N* = 1,035).

Characteristic	Total (*N* = 1,035)	Female (*n* = 541)	Male (*n* = 494)
Age group (years)			
18–24	334 (32.3%)	178 (32.9%)	156 (31.6%)
25–64	695 (67.1%)	358 (66.2%)	337 (68.2%)
>65	6 (0.6%)	5 (0.9%)	1 (0.2%)
Residence			
Urban	858 (82.9%)	437 (80.8%)	421 (85.2%)
Rural	177 (17.1%)	104 (19.2%)	73 (14.8%)
Education level			
Primary or less	54 (5.2%)	31 (5.7%)	23 (4.7%)
Secondary school	194 (18.8%)	98 (18.1%)	96 (19.4%)
Bachelor’s degree	438 (42.3%)	224 (41.4%)	214 (43.3%)
Master’s degree or above	349 (33.7%)	188 (34.8%)	161 (32.6%)
Monthly income (Moroccan Dirham = MAD)			
<4,000	553 (53.4%)	301 (55.6%)	252 (51.0%)
4,000–8,000	306 (29.6%)	150 (27.7%)	156 (31.6%)
8,001–12,000	93 (9.0%)	44 (8.1%)	49 (9.9%)
12,001–16,000	51 (4.9%)	30 (5.5%)	21 (4.3%)
>16,000	32 (3.1%)	16 (3.0%)	16 (3.2%)
BMI category (kg/m^2^)^*^			
Underweight/Normal (<25)	405 (39.1%)	241 (44.5%)	164 (33.2%)
Overweight (25–29.9)	470 (45.4%)	216 (39.9%)	254 (51.4%)
Obese (≥30)	160 (15.5%)	84 (15.5%)	76 (15.4%)

^*^BMI calculated from self-reported weight and height groups.

### 3.2 Dietary practices and consumption frequencies

The frequency of various dietary practices among participants is detailed in Table 2. The study revealed contrasting patterns. Positive practices were highly prevalent: a large majority of the participants reported often or always consuming fresh fruits (89.7%), fresh vegetables (89.0%), and fiber-rich foods (85.6%). However, the consumption of potentially unhealthy foods was also common, with around 76.6% reporting an often or always consumption of foods rich in fat, and nearly half (49.1%) reporting the same frequency for consuming sweetened beverages. The practice of adding salt to food after cooking was relatively uncommon, with 49.7% reporting never or rarely doing so.

**Table 2:** Dietary practices of the study participants (*N* = 1,035).

Food consumption	Never	Rarely	Sometimes	Often	Always	Mean Score (±SD)
Consume fresh fruits	0.2%	4.3%	5.8%	49.0%	40.7%	4.26 (±0.77)
Consume fresh vegetables	0.6%	5.1%	5.3%	52.7%	36.3%	4.19 (±0.80)
Drink sweetened beverages	5.8%	33.0%	12.1%	31.1%	18.0%	2.78 (±1.24)
Add salt after cooking	22.2%	27.5%	29.2%	12.9%	8.1%	2.43 (±1.20)
Consume fiber-rich foods	0.8%	8.0%	5.6%	45.3%	40.3%	4.16 (±0.91)
Consume fried foods	3.2%	36.1%	28.4%	29.5%	2.8%	2.07 (±0.94)
Consume high-fat foods	1.0%	8.3%	14.1%	57.9%	18.7%	3.85 (±0.85)
Try to reduce sugar intake	23.9%	32.0%	30.9%	9.0%	4.3%	2.62 (±1.07)

Regarding meal frequency, the majority of participants (66.7%) reported consuming three meals per day, which aligns with traditional eating patterns. However, a significant proportion (18.7%) reported eating more than three meals daily, while 14.4% consumed only two meals. Only a minimal fraction of the sample (0.2%) reported eating just one meal per day.

### 3.3 History of dieting and use of dietary monitoring tools

**Figure 1:**
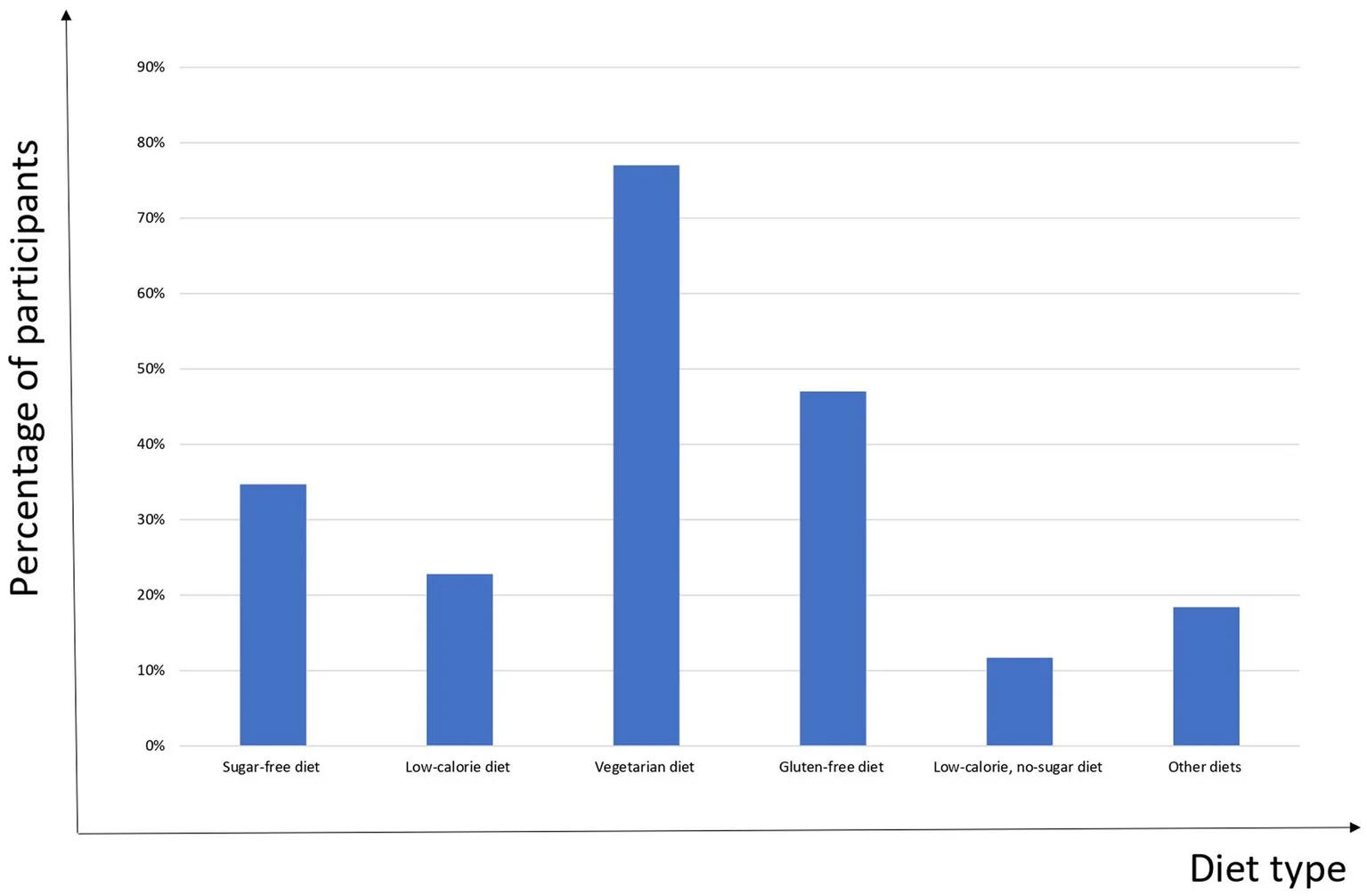
Most prevalent types of diets followed by participants (*n* = 530).

Nearly half of the participants (49.6%) reported having followed a diet to improve their health. Among these respondents (*n* = 530), the most frequently reported types of diets were sugar-free (34.7%), low-calorie (22.8%), and vegetarian (7.7%) diets (Figure 1). Despite this engagement with dietary modification, the use of technology for monitoring was very low; only 9.5% of the total sample reported using applications or tools to track their dietary intake or calorie consumption.

### 3.4 Associations between dietary habits and sociodemographic factors

The chi-square analysis revealed several statistically significant associations between dietary habits and sociodemographic factors. After applying the Bonferroni correction for multiple comparisons (α_adj = 0.001), the following associations remained significant: adding salt after cooking with education (*p* < 0.001, *V* = 0.126), and consuming fried foods with education (*p* < 0.001, *V* = 0.12), and trying to reduce sugar intake with age (*p* < 0.001, *V* = 0.14). Other associations reported as nominally significant (*p* < 0.05) should be interpreted with caution, as they may be due to Type I error. The findings should be considered exploratory, and confirmatory studies are needed to validate these associations.

Fresh fruit consumption showed nominally significant associations with gender (*C* = 0.102, *p* = 0.03) and residence (*C* = 0.104, *p* = 0.02), but these did not survive Bonferroni correction. Sweetened beverage consumption was nominally significantly associated with age (*C* = 0.126, *p* = 0.03) and gender (*C* = 0.138, *p* < 0.001), with gender representing one of the strongest associations observed, though only the gender association approached significance after correction. Similarly, fiber-rich food consumption was nominally associated with gender (*C* = 0.112, *p* = 0.01).

Stronger associations were observed for certain dietary behaviors. Adding salt after cooking demonstrated the strongest relationship overall, with education level (*C* = 0.215, *p* < 0.001), which remained significant after Bonferroni correction. Fried food consumption also revealed multiple associations, being nominally significantly related to age (*C* = 0.152, *p* = 0.002), gender (*C* = 0.132, *p* = 0.001), and education level (*C* = 0.196, *p* < 0.001), with the education association remaining significant after correction. High-fat food consumption showed weaker, yet nominally significant, associations with gender (*C* = 0.096, *p* = 0.05) and residence (*C* = 0.114, *p* = 0.009). Attempts to reduce sugar intake were significantly linked to both age (*C* = 0.187, *p* < 0.001) and monthly income (*C* = 0.170, *p* = 0.01), with the association with age remaining significant after correction.

When examining the independent associations between sociodemographic factors and dietary behaviors using multivariable multinomial logistic regression (adjusting for all other sociodemographic variables simultaneously), several patterns emerged. Females had significantly higher odds of consuming fresh fruits ‘Often/Always’ compared to males (adjusted OR = 1.48, 95% CI: 1.05–2.08, *p* = 0.03), and this association remained significant after adjustment for age, education, residence, and income. Males had higher odds of drinking sweetened beverages ‘Often/Always’ (adjusted OR = 1.72, 95% CI: 1.24–2.38, *p* < 0.001). Higher education was independently associated with lower odds of adding salt after cooking (Bachelor’s: adjusted OR = 0.48, 95% CI: 0.32–0.72, *p* < 0.001; Master’s+: adjusted OR = 0.39, 95% CI: 0.25–0.61, *p* < 0.001). Age ≥25 years was associated with higher odds of trying to reduce sugar intake (adjusted OR = 1.45, 95% CI: 1.08–1.95, *p* = 0.02). Urban residence was independently associated with higher consumption of high-fat foods (adjusted OR = 1.38, 95% CI: 1.02–1.87, *p* = 0.04). After adjusting for other variables, income was not independently associated with any dietary behavior (see Table 3).

**Table 3:** Significant chi-square (*χ*^2^) associations between dietary habits and sociodemographic factors after Bonferroni correction (*p* < 0.001).

Dietary habit	Age	Gender	Education	Residence	Income
Consume fresh fruits	ns	ns	ns	ns	ns
Consume fresh vegetables	ns	ns	ns	ns	ns
Drink sweetened beverages	ns	ns	ns	ns	ns
Add salt after cooking	ns	ns	*χ*^2^ = 49.9, *p* < 0.001, *V* = 0.126	ns	ns
Consume fiber-rich foods	ns	ns	ns	ns	ns
Consume fried foods	ns	ns	*χ*^2^ = 41.2, *p* < 0.001, *V* = 0.12	ns	ns
Consume high-fat foods	ns	ns	ns	ns	ns
Try to reduce sugar intake	*χ*^2^ = 37.3, *p* < 0.001, *V* = 0.14	ns	ns	ns	ns

ns, not significant after Bonferroni correction (*p* ≥ 0.001); *V* = Cramér’s *V*.

Notable patterns emerged from these findings. Gender appeared as the most consistent predictor of dietary habits, showing significant associations with five out of the eight dietary behaviors examined. Age and education level each influenced three dietary behaviors, while residence and monthly income were linked to two behaviors. No statistically significant associations were observed between any dietary habits and BMI. Overall, contingency coefficients ranged from 0.096 to 0.215, suggesting weak to moderate associations. To further explore whether associations differed by participant characteristics, we performed gender-stratified and residence-stratified chi-square analyses (Supplementary Tables S1, S2). In gender-stratified analyses, associations between sweetened beverage consumption and age remained significant only among females (*p* = 0.04, *V* = 0.10), while fried food consumption was associated with age in both sexes but more strongly in males (*p* = 0.008, V = 0.12 in females; *p* = 0.01, *V* = 0.11 in males). In urban–rural stratified analyses, several associations that were significant in the overall sample lost significance after stratification, suggesting that the overall effects were driven primarily by urban participants. Detailed results, including all *p*-values and Cramér’s *V*, are provided in Supplementary Tables S1, S2. Although statistically significant, these sociodemographic factors account for only a limited proportion of the variance in dietary behaviors (see Table 4).

**Table 4:** Nominally significant chi-square (*χ*^2^) associations between dietary habits and sociodemographic factors (*p* < 0.05, but *p* ≥ 0.001).

Dietary habit	Age	Gender	Education	Residence	Income
Consume fresh fruits	ns	*χ*^2^ = 10.9, *p* = 0.03, *V* = 0.10	ns	*χ*^2^ = 11.4, *p* = 0.02, *V* = 0.11	ns
Consume fresh vegetables	ns	ns	ns	ns	ns
Drink sweetened beverages	*χ*^2^ = 16.8, *p* = 0.03, *V* = 0.09	*χ*^2^ = 20.0, *p* < 0.001, *V* = 0.14	ns	ns	ns
Add salt after cooking	ns	ns	*χ*^2^ = 49.9, *p* = 0.001, *V* = 0.126	ns	ns
Consume fiber-rich foods	ns	*χ*^2^ = 13.0, *p* = 0.01, *V* = 0.11	ns	ns	ns
Consume fried foods	*χ*^2^ = 24.4, *p* = 0.002, *V* = 0.11	*χ*^2^ = 18.4, *p* = 0.001, V = 0.13	*χ*^2^ = 41.2, *p* = 0.001, *V* = 0.12	ns	ns
Consume high-fat foods	ns	*χ*^2^ = 9.7, *p* = 0.05, *V* = 0.09	ns	*χ*^2^ = 13.6, *p* = 0.009, *V* = 0.12	ns
Try to reduce sugar intake	*χ*^2^ = 37.3, *p* = 0.001, *V* = 0.14	ns	ns	ns	*χ*^2^ = 30.8, *p* = 0.01, *V* = 0.09

ns, not significant (*p* ≥ 0.05); *V* = Cramér’s *V*. Associations in Table 3 remained significant after Bonferroni correction.

### 3.5 Multivariable logistic regression for overweight/obesity

The multivariable logistic regression model (Table 5) demonstrated acceptable goodness of fit as assessed by the Hosmer-Lemeshow test (*χ*^2^ = 8.32, df = 8, *p* = 0.40), indicating that the model adequately fits the observed data (i.e., no significant discrepancy between observed and predicted values). The model’s discriminative ability was moderate, with an Area Under the Receiver Operating Characteristic Curve (AUC) of 0.71 (95% CI: 0.68–0.74), suggesting acceptable to good discrimination between overweight/obese and non-overweight participants. The model’s overall fit was further supported by AIC = 1232.03, BIC = 1330.87, and McFadden’s pseudo *R*^2^ = 0.08. Classification accuracy was 66.3% overall (sensitivity: 68.2%, specificity: 63.0%, positive predictive value: 74.1%, negative predictive value: 56.2%).

**Table 5:** Multivariable logistic regression analysis for overweight/obesity (BMI ≥ 25).

Variable	Category (reference)	Adjusted OR	95% CI	*p*-value
Age (ref: 18-24)	25–64	3.69	(2.55–5.34)	<0.001
	>65	6.28	(0.99–39.68)	0.05
Gender (ref: Female)	Male	0.98	(0.73–1.31)	0.87
Residence (ref: Rural)	Urban	1.14	(0.78–1.66)	0.51
Education (ref: Bachelor’s degree)	Master’s degree or above	0.85	(0.61–1.19)	0.34
	Primary	2.06	(0.68–6.27)	0.20
	Secondary high school	1.41	(0.99–2.14)	0.11
	Secondary middle school	6.29	(2.97–13.34)	<0.001
Income (ref: 12,001–16,000 MAD)	4,000–8,000	0.67	(0.35–1.28)	0.23
	8,001–12,000	0.81	(0.39–1.67)	0.56
	<4,000	0.73	(0.38–1.41)	0.35
	>16,000	1.06	(0.43–2.64)	0.99
Dietary behavior scores (continuous, 1–5)	Fruits score	1.04	(0.85–1.27)	0.73
	Vegetables score	1.10	(0.91–1.34)	0.33
	Sweetened beverages score	0.91	(0.80–1.03)	0.14
	Salt after cooking score	0.99	(0.88–1.12)	0.89
	Fiber-rich foods score	0.79	(0.67–0.93)	0.004
	Fried foods score	1.21	(1.01–1.43)	0.04
	High-fat foods score	1.04	(0.87–1.25)	0.65

Model fit: AIC = 1230.45, BIC = 1320.18, McFadden’s pseudo *R*^2^ = 0.08; Hosmer-Lemeshow *χ*^2^ = 7.98, df = 8, *p* = 0.44; AUC = 0.71 (95% CI: 0.68–0.74).

## 4 Discussion

The socio-demographic profile of our sample predominantly urban (82.9%), highly educated (79.8% with bachelor’s degree or higher), and with a mean age in the 25–64 years categoryrequires careful consideration when interpreting the findings. While this composition is consistent with the digital recruitment strategy employed, it diverges substantially from the general Moroccan population profile. According to the 2024 Population and Housing Census, only 62.8% of Moroccans reside in urban areas, and the proportion of university graduates remains below 30% nationally. Thus, our findings are not representative of the Moroccan adult population as a whole. Rather, they provide insights into the dietary behaviors and technology-related practices of a specific, emerging demographic segment: urban, educated, and digitally connected Moroccans. This population is of particular interest because it is at the forefront of dietary transitions and represents a key target group for digital health interventions. However, the extent to which these findings apply to rural populations, less-educated individuals, or older adults remains unknown and should be the subject of future research with more representative sampling strategies. The high prevalence of overweight/obesity observed in this educated sample (60.9%) is particularly noteworthy, as it challenges the traditional inverse association between education and obesity often observed in high-income countries, suggesting a nutrition transition pattern more characteristic of middle-income settings where higher socioeconomic status is increasingly associated with obesity risk (7, 22, 23).

A key finding is the paradoxical nature of dietary practices. High frequencies of fresh fruit, vegetable, and fiber consumption indicate the persistence of positive elements of the traditional MeDi (7, 22). However, the equally high consumption of high-fat foods and sweetened beverages points to a significant departure from this pattern. Our findings suggest a complex nutritional landscape characterized by the coexistence of traditional and modern dietary practices, consistent with patterns observed during nutrition transitions in other middle-income countries (24, 25). However, as this is a cross-sectional study, we cannot directly confirm a temporal transition in dietary patterns. The observed patterns are suggestive of a nutrition transition but require longitudinal studies for confirmation. This “dual burden” of dietary behavior has been observed in other transitioning economies, where the influx of processed, energy-dense foods complements rather than completely displaces traditional food habits (7). The common report of consuming three meals a day suggests resilience in traditional meal structures, but the content of these meals appears to be changing.

Nearly half of the participants (49.6%) reported having followed a diet, primarily sugar-free or low-calorie. This suggests a stated interest in dietary modification or weight control, which may reflect increased awareness of non-communicable diseases (NCDs) (26). However, the very low use of dietary monitoring technology (9.5%) is striking, especially given the high education level of the sample. This suggests a significant gap between the intention to eat healthily and the use of digital tools to facilitate this goal. This contrasts with trends in high-income countries where app usage for health monitoring is more widespread (27), but may be similar to other middle-income settings where cultural preferences for informal advice or barriers to digital health adoption persist (28). The low utilization of technology for dietary monitoring, despite a stated interest in dietary modification, suggests a potential gap that may warrant further investigation. However, it is important to note that this observation is based on cross-sectional data and does not imply a causal relationship between technology use and dietary behaviors. Alternative explanations for this low uptake include: (1) lack of awareness of available technologies among the Moroccan population; (2) cultural preferences for traditional advice sources (family, friends, healthcare providers) over digital tools; (3) concerns about data privacy and security; (4) perceived complexity or lack of user-friendliness of existing applications; (5) cost barriers for premium features; (6) language barriers (most applications are in English or French, while Arabic is the primary language for many Moroccans); and (7) limited availability of Moroccan-specific food databases for accurate calorie counting. Future qualitative and mixed-methods research would be valuable to explore these potential explanations and to inform the development of culturally adapted digital health interventions that address the specific needs and preferences of Moroccan users.

When examining the independent associations between sociodemographic factors and dietary behaviors using multivariable multinomial logistic regression (adjusting for all other sociodemographic variables simultaneously), gender emerged as the most consistent independent predictor. Gender was associated with five of the eight dietary practices, a finding echoed in studies from both high and low-middle-income countries that highlight gendered roles in food choice and preparation (29). The strong association between higher education and the practice of adding salt after cooking (C = 0.215) is intriguing and may reflect greater awareness of hypertension risks, leading to more conscious, albeit not always accurate, salt-use behaviors (30). After adjusting for other factors, the association with education remained significant, suggesting that education independently influences salt-related behaviors beyond other sociodemographic factors. The absence of any significant association between dietary habits and BMI is consistent with some studies (31) and underscores the multifactorial etiology of obesity, involving genetic, metabolic, physical activity, and environmental factors not captured in this study.

### 4.1 Limitations

Several limitations warrant careful consideration when interpreting our findings. First, the convenience sampling method via social media platforms inevitably introduced selection bias. The sample over-represents urban, university-educated, and digitally literate individuals, while under-representing rural populations, those with lower educational attainment, and older adults who are less active on social media. Consequently, the findings cannot be generalized to the entire Moroccan adult population. Instead, they should be interpreted as applying primarily to urban, educated, digitally connected Moroccan adults. Second, the reliance on self-reported anthropometric data is susceptible to systematic misreporting, with individuals tending to underestimate weight and overestimate height a phenomenon well-documented in obesity research. This may have led to an underestimation of overweight and obesity prevalence in our sample. While categorical reporting of weight and height groups was employed to reduce respondent burden, this approach introduces additional measurement error through the use of midpoints for BMI calculation, potentially misclassifying individuals who fall near category boundaries. Third, the dietary assessment was based on a limited set of eight self-reported frequency items, which, while adapted from validated instruments and demonstrating acceptable internal consistency (Cronbach’s *α* = 0.78), does not capture the full complexity of dietary patterns, portion sizes, cooking methods, or overall dietary quality. This limitation may explain the absence of associations between some dietary behaviors and BMI in our bivariate analyses. Fourth, the cross-sectional design precludes the establishment of causal or temporal relationships between dietary behaviors, technology use, and sociodemographic factors. For instance, we cannot determine whether observed associations reflect long-standing patterns or recent changes in lifestyle, nor can we infer causality between dietary behaviors and obesity status. Fifth, residual confounding remains a concern despite the inclusion of multiple sociodemographic factors and dietary variables in our multivariable model. Unmeasured confounders such as physical activity levels, sleep patterns, psychological stress, family history of obesity, health literacy, cultural attitudes toward body image, and access to healthcare services may have influenced both dietary behaviors and obesity status. Sixth, the study did not collect information on medication use (e.g., corticosteroids, antidepressants), comorbidities (e.g., hypothyroidism, diabetes), or pregnancy status, which could affect body weight and dietary patterns. Seventh, the lack of qualitative data on participants’ experiences with, or reasons for non-use of, nutrition-monitoring technologies limits our understanding of the barriers to adoption. We cannot determine whether non-use reflects lack of awareness, cost barriers, privacy concerns, perceived irrelevance, or other factors. Eighth, social desirability bias may have influenced responses to dietary questions, with participants potentially overreporting healthy behaviors and underreporting unhealthy practices, leading to an overly optimistic picture of dietary practices. Ninth, the online survey format may have excluded individuals with limited internet access or digital literacy, further contributing to the selection bias already discussed. Tenth, age was collected using broad categories (18–24, 25–64, ≥65), which prevented more detailed analysis of dietary behaviors across narrower age bands. Future studies should use continuous age or finer categories to capture age-related nuances. Finally, the Bonferroni correction for multiple comparisons, while necessary to control Type I error, is conservative and may have led to Type II errors (i.e., missing true associations). Therefore, nominally significant findings (*p* < 0.05 but *p* ≥ 0.001) should not be dismissed but rather interpreted as hypothesis-generating and requiring confirmation in future studies with larger sample sizes.

## 5 Conclusion

This cross-sectional study provides a contemporary descriptive account of dietary behaviors, technology use, and their socio-demographic correlates among a convenience sample of urban, educated Moroccan adults. The findings reveal a complex nutritional landscape characterized by a coexistence of traditional healthy dietary elements (high fruit, vegetable, and fiber consumption) with less favorable patterns associated with globalization and dietary transitions (frequent consumption of high-fat foods and sweetened beverages). The high prevalence of overweight and obesity (60.9%) in this specific population subgroup points to a public health concern that merits attention, although the generalizability of this finding to the broader Moroccan population remains uncertain given the non-probability sampling method employed.

Regarding digital nutrition-monitoring technologies, our findings indicate remarkably low uptake (9.5%) despite a stated interest in dietary modification. This observation suggests a potential area for further investigation, but does not establish causation or justify immediate implementation of digital health interventions. The gap between intention and behavior in technology adoption warrants systematic exploration through qualitative research methods.

Given the cross-sectional design and the limitations of convenience sampling, the associations identified between sociodemographic factors and dietary behaviors should be interpreted as exploratory rather than confirmatory. The fact that gender, education, and age were significant associated factors suggests, but does not prove, that targeted rather than one-size-fits-all nutritional interventions may be more effective. However, this hypothesis requires testing through longitudinal studies and randomized controlled trials before translation into public health policy.

A key strength of this study is its relatively large sample size and the use of a validated questionnaire adapted to the Moroccan context. It provides much-needed recent data on a population subgroup at the forefront of dietary transition. However, the findings must be interpreted considering the limitations discussed above, particularly the selection bias from convenience sampling, the use of self-reported data, and the cross-sectional design that precludes causal inferences.

Hypothesis-generating suggestions emerging from this study include the potential value of culturally adapted digital health interventions that address the specific needs and preferences of Moroccan users. Such interventions might incorporate: (a) local dietary databases with Moroccan foods; (b) Arabic language interfaces; (c) integration with social and community support features; (d) simplified user interfaces accessible to individuals with varying levels of digital literacy; and (e) affordability through free or low-cost models. However, qualitative and co-design studies are first needed to understand why nutrition-monitoring technologies remain underused, what features would enhance their acceptability, and how they could be integrated with existing dietary practices in the Moroccan cultural context. Rigorous evaluation through randomized controlled trials would be necessary before any large-scale implementation. Additionally, future research should employ longitudinal designs and more representative sampling strategies to better understand the causal pathways leading to obesity in the Moroccan context and to evaluate the effectiveness of tailored public health programs aimed at promoting the preservation of healthy traditional diets while mitigating the adoption of unhealthy modern food habits.

In conclusion, within the limits of our study design and sampling strategy, we observed that urban Moroccan adults in our sample exhibit dietary patterns indicative of a nutrition transition, with a high prevalence of overweight. The low use of nutrition-monitoring technologies, despite a stated interest in dieting, highlights a potential avenue for future research and intervention development. However, cautious interpretation is warranted, and confirmatory studies using probability sampling, objective anthropometric measurements, and longitudinal designs are needed to validate these findings and to guide evidence-based public health recommendations in Morocco.

## Data Availability

The original contributions presented in the study are included in the article/Supplementary material, further inquiries can be directed to the corresponding authors.
