# Supplementary material for: Dietary behaviors, nutrition-monitoring technology use, and their socio-demographic determinants among Moroccan adults: a cross-sectional study
Source: Front Nutr. 2026 Sep 10;13:1912937. doi: 10.3389/fnut.2026.1912937 (PMC13603027; doi:10.3389/fnut.2026.1912937)
Supplement: Supplementary file 1 [file Table_1.docx]

**Supplementary Table S1.** Gender-stratified associations between dietary habits and selected sociodemographic factors (χ², p-value, Cramér's V). Associations with *p* < 0.05 are highlighted in bold; however, only associations with p < 0.001 survived Bonferroni correction.

**Females (n=541)**

|  | Females (n=541) | | | |  | | |
| --- | --- | --- | --- | --- | --- | --- | --- |
| Dietary habit | **Age (χ², p, V)** | **Education (χ², p, V)** | **Residence (χ², p, V)** | **Income (χ², p, V)** | | |  |
| Consume fresh fruits | χ²=9.8, p=0.12, V=0.09 | χ²=7.2, p=0.45, V=0.07 | χ²=12.4, p=0.03, V=0.11 | χ²=8.5, p=0.68, V=0.06 | | |  |
| Consume fresh vegetables | χ²=5.6, p=0.68, V=0.05 | χ²=11.3, p=0.08, V=0.08 | χ²=4.2, p=0.61, V=0.05 | χ²=10.1, p=0.42, V=0.07 | | |  |
| Drink sweetened beverages | χ²=14.2, p=0.04, V=0.10 | χ²=16.8, p=0.02, V=0.09 | χ²=10.5, p=0.08, V=0.08 | χ²=15.3, p=0.06, V=0.08 | | |  |
| Add salt after cooking | χ²=6.3, p=0.52, V=0.06 | χ²=28.9, p<0.001, V=0.14 | χ²=8.1, p=0.19, V=0.07 | χ²=11.2, p=0.12, V=0.08 | | |  |
| Consume fiber-rich foods | χ²=8.7, p=0.26, V=0.07 | χ²=19.4, p=0.01, V=0.10 | χ²=6.8, p=0.44, V=0.06 | χ²=9.6, p=0.38, V=0.07 | | |  |
| Consume fried foods | χ²=16.5, p=0.008, V=0.12 | χ²=32.1, p<0.001, V=0.13 | χ²=7.4, p=0.31, V=0.07 | χ²=10.8, p=0.18, V=0.08 | | |  |
| Consume high-fat foods | χ²=5.2, p=0.71, V=0.05 | χ²=15.6, p=0.03, V=0.09 | χ²=13.2, p=0.02, V=0.11 | χ²=8.3, p=0.52, V=0.07 | | |  |
| Try to reduce sugar intake | χ²=31.8, p<0.001, V=0.15 | χ²=17.3, p=0.02, V=0.09 | χ²=9.5, p=0.27, V=0.07 | χ²=19.7, p=0.02, V=0.10 | | |  |
| Males (n=494) | | | | | | |  |
| Dietary habit | **Age (χ², p, V)** | **Education (χ², p, V)** | **Residence (χ², p, V)** | **Income (χ², p, V)** | |  |  |
| Consume fresh fruits | χ²=10.2, p=0.09, V=0.08 | χ²=6.8, p=0.52, V=0.07 | χ²=11.8, p=0.06, V=0.09 | χ²=7.6, p=0.71, V=0.06 | |  |  |
| Consume fresh vegetables | χ²=4.8, p=0.77, V=0.05 | χ²=10.5, p=0.12, V=0.08 | χ²=3.9, p=0.83, V=0.04 | χ²=9.2, p=0.49, V=0.07 | |  |  |
| Drink sweetened beverages | χ²=13.6, p=0.08, V=0.09 | χ²=18.2, p=0.03, V=0.10 | χ²=11.1, p=0.10, V=0.08 | χ²=14.8, p=0.07, V=0.09 | |  |  |
| Add salt after cooking | χ²=7.8, p=0.39, V=0.07 | χ²=35.4, p<0.001, V=0.16 | χ²=8.6, p=0.34, V=0.07 | χ²=10.4, p=0.20, V=0.08 | |  |  |
| Consume fiber-rich foods | χ²=9.1, p=0.33, V=0.07 | χ²=20.8, p=0.02, V=0.11 | χ²=6.2, p=0.57, V=0.06 | χ²=8.9, p=0.47, V=0.07 | |  |  |
| Consume fried foods | χ²=17.8, p=0.01, V=0.11 | χ²=34.6, p<0.001, V=0.14 | χ²=7.9, p=0.42, V=0.07 | χ²=11.5, p=0.26, V=0.08 | |  |  |
| Consume high-fat foods | χ²=5.8, p=0.58, V=0.06 | χ²=14.2, p=0.05, V=0.09 | χ²=14.5, p=0.03, V=0.11 | χ²=8.7, p=0.48, V=0.07 | |  |  |
| Try to reduce sugar intake | χ²=29.4, p<0.001, V=0.14 | χ²=18.7, p=0.03, V=0.10 | χ²=8.3, p=0.44, V=0.06 | χ²=20.1, p=0.03, V=0.10 | |  |  |
| Note: χ² = chi-square statistic; df = degrees of freedom (not shown for brevity); V = Cramér's V. Significant associations (p < 0.05) are highlighted in bold; however, only associations with p < 0.001 survived Bonferroni correction (α_adjusted = 0.001). | | | | | |  |  |

**Supplementary Table S2.** Residence-stratified associations between dietary habits and sociodemographic factors (χ², p-value, Cramér's V). Associations with p < 0.05 are highlighted in bold; however, only associations with *p* < 0.001 survived Bonferroni correction.

**Urban (n=858)**

| Dietary habit | Age (χ², p, V) | Gender (χ², p, V) | Education (χ², p, V) | Income (χ², p, V) |
| --- | --- | --- | --- | --- |
| Consume fresh fruits | χ²=11.4, p=0.08, V=0.07 | χ²=13.8, p=0.04, V=0.09 | χ²=8.2, p=0.58, V=0.06 | χ²=7.9, p=0.69, V=0.06 |
| Consume fresh vegetables | χ²=6.8, p=0.71, V=0.05 | χ²=8.5, p=0.32, V=0.06 | χ²=14.6, p=0.10, V=0.08 | χ²=11.3, p=0.44, V=0.07 |
| Drink sweetened beverages | χ²=17.4, p=0.03, V=0.10 | χ²=24.8, p<0.001, V=0.13 | χ²=19.2, p=0.03, V=0.09 | χ²=15.8, p=0.07, V=0.08 |
| Add salt after cooking | χ²=7.2, p=0.48, V=0.06 | χ²=10.6, p=0.12, V=0.07 | χ²=38.6, p<0.001, V=0.12 | χ²=13.4, p=0.15, V=0.08 |
| Consume fiber-rich foods | χ²=9.8, p=0.36, V=0.07 | χ²=15.2, p=0.02, V=0.10 | χ²=22.4, p=0.01, V=0.10 | χ²=10.8, p=0.43, V=0.07 |
| Consume fried foods | χ²=22.1, p=0.005, V=0.11 | χ²=26.3, p=0.003, V=0.12 | χ²=41.8, p<0.001, V=0.13 | χ²=14.2, p=0.22, V=0.08 |
| Consume high-fat foods | χ²=6.5, p=0.61, V=0.06 | χ²=12.8, p=0.06, V=0.08 | χ²=20.6, p=0.02, V=0.10 | χ²=9.6, p=0.47, V=0.07 |
| Try to reduce sugar intake | χ²=33.6, p<0.001, V=0.14 | χ²=4.2, p=0.89, V=0.03 | χ²=21.8, p=0.03, V=0.09 | χ²=28.4, p=0.02, V=0.10 |

**Rural (n=177)**

| Dietary habit | Age (χ², p, V) | Gender (χ², p, V) | Education (χ², p, V) | Income (χ², p, V) |
| --- | --- | --- | --- | --- |
| Consume fresh fruits | χ²=8.9, p=0.41, V=0.08 | χ²=7.6, p=0.27, V=0.09 | χ²=6.4, p=0.62, V=0.07 | χ²=8.1, p=0.53, V=0.08 |
| Consume fresh vegetables | χ²=5.4, p=0.83, V=0.06 | χ²=5.9, p=0.58, V=0.07 | χ²=10.2, p=0.34, V=0.09 | χ²=7.3, p=0.67, V=0.07 |
| Drink sweetened beverages | χ²=12.6, p=0.12, V=0.10 | χ²=14.8, p=0.04, V=0.12 | χ²=13.5, p=0.09, V=0.11 | χ²=11.9, p=0.14, V=0.10 |
| Add salt after cooking | χ²=7.1, p=0.59, V=0.07 | χ²=8.4, p=0.22, V=0.09 | χ²=29.8, p<0.001, V=0.18 | χ²=12.4, p=0.19, V=0.10 |
| Consume fiber-rich foods | χ²=8.3, p=0.47, V=0.08 | χ²=12.1, p=0.08, V=0.11 | χ²=16.8, p=0.04, V=0.13 | χ²=9.2, p=0.52, V=0.08 |
| Consume fried foods | χ²=14.8, p=0.06, V=0.12 | χ²=16.2, p=0.03, V=0.13 | χ²=32.4, p<0.001, V=0.17 | χ²=11.6, p=0.23, V=0.10 |
| Consume high-fat foods | χ²=6.2, p=0.71, V=0.07 | χ²=10.4, p=0.09, V=0.11 | χ²=15.2, p=0.04, V=0.13 | χ²=8.8, p=0.49, V=0.08 |
| Try to reduce sugar intake | χ²=26.4, p<0.01, V=0.15 | χ²=6.8, p=0.63, V=0.07 | χ²=14.6, p=0.06, V=0.12 | χ²=18.7, p=0.04, V=0.13 |
| Note: χ² = chi-square statistic; df = degrees of freedom (not shown for brevity); V = Cramér's V. Significant associations (p < 0.05) are highlighted in bold; however, only associations with p < 0.001 survived Bonferroni correction (α_adjusted = 0.001). Rural subgroup (n=177) has limited statistical power; results should be interpreted with caution. | | | | |

**Supplementary Table S3.** Minimum Expected Cell Frequencies for Chi-Square Analyses of Dietary Behaviors by Sociodemographic Factors (N=1,035)

| Dietary Behavior | Age | Gender | Education | Residence | Income |
| --- | --- | --- | --- | --- | --- |
| Consume fresh fruits | 8.7 | 12.4 | 6.3 | 14.2 | 5.8 |
| Consume fresh vegetables | 9.1 | 13.1 | 6.8 | 15.0 | 6.2 |
| Drink sweetened beverages | 11.3 | 15.8 | 8.2 | 18.1 | 7.5 |
| Add salt after cooking | 10.2 | 14.3 | 7.5 | 16.5 | 6.9 |
| Consume fiber-rich foods | 8.5 | 11.9 | 6.1 | 13.8 | 5.6 |
| Consume fried foods | 12.8 | 17.2 | 9.4 | 19.7 | 8.3 |
| Consume high-fat foods | 9.7 | 13.5 | 7.1 | 15.8 | 6.5 |
| Try to reduce sugar intake | 10.9 | 15.1 | 7.9 | 17.3 | 7.2 |

**Note:** All minimum expected cell frequencies exceed the conventional threshold of 5.0, confirming that the assumptions of the chi-square test are met for all analyses. The lowest minimum expected frequency was 5.6 (fiber-rich foods × income), well above the acceptable limit.
